# Positivity rates of SAR-CoV-2 infection in orthodontic patients at the orthodontic clinic, University of Illinois Chicago

**DOI:** 10.1371/journal.pone.0270311

**Published:** 2022-06-23

**Authors:** Phimon Atsawasuwan, Dhammacari Martin Del Campo, Laura Martin Del Campo, Grace Viana, Sriram Ravindran, Veerasathpurush Allareddy, Shrihari Kadkol

**Affiliations:** 1 Department of Orthodontics, College of Dentistry, University of Illinois Chicago, Chicago, Illinois, United States of America; 2 Department of Oral Biology, College of Dentistry, University of Illinois Chicago, Chicago, Illinois, United States of America; 3 Department of Pathology, College of Medicine, University of Illinois Chicago, Chicago, Illinois, United States of America; Virginia Commonwealth University, UNITED STATES

## Abstract

COVID-19 has impacted and increased risks for healthcare providers, including orthodontists. There is no information regarding the potential transmission risks in the orthodontic community. This study aims to compare the positivity rate of SARS-CoV-2 infection in orthodontic patients at the University of Illinois Chicago (UIC) orthodontic clinic to the positivity rate of the local population in Chicago. All orthodontic patients who sought treatment at the UIC orthodontic clinic from June 16 to October 31, 2021, were invited to participate in the study. Three milliliters of saliva from the participants were collected in the sample collection tubes and subjected to a polymerase chain reaction (PCR) based assay to detect SAR-CoV-2. All participants’ age, sex, history of COVID-19 infection, and vaccination status were recorded. The COVID-19 positivity rates of Chicago, Cook County of Illinois, and the orthodontic clinic at UIC were compared. One thousand four hundred and thirty-seven orthodontic patients aged 6 to 70 years old (41.8% males and 58.2% females) participated in the study. Among all participants, nine participants tested positive for SARS-CoV-2 (5 males and 4 females). During the study, the average COVID-19 positivity rate at the UIC orthodontic clinic was 0.626%. All of the positive participants were asymptomatic, and two of the participants had a history of COVID-19 infection. Among all positive participants, three participants had received complete COVID-19 vaccination. An increased frequency of positive cases at the orthodontic clinic was observed during the time of high positivity rate in Chicago and Cook County. A potential risk of COVID-19 transmission from patients to orthodontic providers remains, even with asymptomatic and vaccinated patients.

## Introduction

As of May 20, 2022, 82,820,565 cases of COVID-19, including 998,512 deaths, have been reported in the United States [1]. Transmission of COVID-19 can occur during symptomatic, presymptomatic, and asymptomatic periods. The asymptomatic/presymptomatic transmission of COVID-19 makes the disease highly transmissible, and it is challenging to prevent its transmission [2]. In orthodontic practices, most patients are children and adolescents. Reports showed that children or adolescents frequently exhibited no symptoms despite being infected with SARS-CoV-2 [3–5]. In addition, SARS-CoV-2 viral loads are similar to adults when they have symptoms related to COVID-19 at early stages [6–8]. As such, there is a potential risk of SARS-CoV-2 transmission from patients to orthodontic providers in orthodontic practices. Currently, there is no report or information on the positivity rate of SARS-CoV-2 infection in the orthodontic patient population. Knowing the positivity rate may help initiate proper measures to mitigate the risk of transmission to orthodontic providers.

SARS-CoV-2 is the seventh member of the enveloped, positive-stranded RNA viruses [9]. SARS-CoV-2 can also be detected in saliva [10–12]. The reported salivary SARS-CoV-2 load ranged 10^2^−10^6^ copies/ mL [13,14]. Viral loads in saliva may also be higher than in nasopharyngeal (NP) or oropharyngeal (OP) swabs, increasing the risk of transmission by salivary droplets during coughing, sneezing, or even talking [10–12]. There are 3 approaches to test for SARS-CoV-2; (a) RT-PCR-based tests to detect viral nucleic acid, (b) rapid antigen tests to detect viral proteins, and (c) antibody tests to assess the body’s immune response [15]. Reverse transcription-polymerase chain reaction (RT-PCR) is the gold standard of COVID-19 testing. Besides the NP or OP specimens, the US FDA issued an emergency use authorization (EUA) for saliva as specimens for COVID-19 diagnosis [16–18]. RT-PCR tests are highly sensitive and specific to detecting SARS-CoV-2, with most tests performed on NP/OP swabs [19]. Alternative molecular methods to detect viral nucleic acids, such as RT-LAMP and transcription-mediated amplification, have been reported [20–23].

SARS-CoV-2 can be detected with high sensitivity and specificity in saliva [10–12,16,17,24]. As a specimen type, saliva has many advantages over NP/OP swabs to detect SARS-CoV-2. Saliva can be self-collected, is non-invasive, and does not need a medium to transport to the laboratory [25]. In contrast, collecting NP/OP swabs requires trained personnel, swabs and viral transmitting media [26]. Being non-invasive, saliva can also be obtained multiple times for testing [25]. In addition, the self-collection of saliva reduces the risk of COVID-19 exposure to healthcare personnel during collection [27]. The sensitivity of SARS-CoV-2 detection in the saliva is comparable to that of NP/OP swabs [18,26]. Several studies have shown that SARS-CoV-2 can be detected in the saliva of asymptomatic persons and outpatients [16,17,28]. In May 2020, the US FDA issued an emergency use authorization (EUA) for tests to detect SARS-CoV-2 in saliva [29]. In Aug 2020, the molecular pathology laboratory in the department of pathology at the University of Illinois Chicago, a CLIA/CAP-accredited laboratory, developed and validated a one-step RT-qPCR test to detect SARS-CoV-2 in saliva. The lab-developed test was used to determine SARS-CoV-2 status in patients enrolled in this study. The test detects the S gene of SARS-CoV-2 with RNaseP as an internal control. The test is highly sensitive and specific and will detect all SARS-CoV-2 variants being monitored, variants of interest, and variants of concern as designated by the Centers for Disease Control and Prevention (CDC). The objective of the study was to evaluate SARS-CoV-2 positivity in the orthodontic patients visiting the orthodontic clinic at the University of Illinois Chicago using the test developed by the molecular pathology laboratory. Knowing the positivity rate of SARS-CoV-2 positivity may better help to assess the risk of SARS-CoV-2 transmission in the orthodontic clinic.

## Materials and methods

### Human subjects and saliva collection

All orthodontic patients who sought orthodontic treatment at the University of Illinois Chicago (UIC) at the orthodontic clinic from June 16 to October 31, 2021, were invited to participate in the study. The study was approved by the Institutional Review Board of the University of Illinois (IRB# 2020–1465). The inclusion criteria included participants aged 7–50 years old who visited the orthodontic clinic for treatment. The participants did not exhibit any COVID-19 symptoms and reported no contact with any COVID-19 patients. The consent form was reviewed and obtained from the eligible participants and their parents/guardians, and the assent form. After initial screening with temperature checking, a set of questionnaires was used to evaluate the demographic information: age, sex, race, COVID-19 vaccination status, and COVID-19 infection history of the patients before the saliva collection. The participants were refrained from eating or drinking 30 mins before the saliva collection and instructed to spit their saliva (5 ml volume) in 50 ml Falcon® tubes with the sample de-identification label, placed in a biohazard bag, and dropped into a storage container. The specimens were transported on the same day for the PCR-based test [30] to the Molecular Pathology laboratory for testing. The results were reported within 24–48 hours. Due to the surveillance purpose, the testing was performed on de-identified specimens, and thus, results are not linked to individual subject. The surveillance testing results cannot be used for individual decision-making or treatment [31]. The COVID-19 positivity rates of Chicago, Cook County, and the orthodontic clinic at UIC were compared on a daily basis during the period of the study.

### SARS-CoV-2 RT-qPCR test

Approximately 200ul of the saliva was extracted on the Kingfisher Flex instrument (Thermofisher) with MagMax Viral/Pathogen isolation kit reagents according to the manufacturer’s protocol. Nucleic acids were eluted in a final volume of 60 μl. 5μl of the extract was analyzed in a one-step RT-qPCR reaction using primers and probes that amplify the S gene of SARS-CoV-2 and the cellular RNaseP gene. RNaseP served as an internal control to ensure adequate specimen collection, nucleic acid extraction, and the absence of RT-PCR inhibition. The primer and probe sequences were (5’>3”): Sgene-F2 AACTCAATTACCCCCTGCATAC, Sgene-R2 TAGTACCATTGGTCCCAGAGACA, Sgene Probe2 HEX TCAGATCCTCAGTTTTACATTCAACTCAGGACTTG BHQ1, RNaseP-F AGATTTGGACCTGCGAGCG, RNaseP-R GAGCGGCTGTCTCCACAAGT, RNaseP probe FAM TTCTGACCTGAAGGCTCTGCGCG BHQ1. Each 25μl reaction contained 6.25μl of 4X TaqPath 1-Step RT-qPCR mix, CG (Thermofisher), 10pmols of Sgene-F2 and R2 primers, 5 pmoles of Sgene Probe2, 0.5pmols of RNaseP-F and R primers and 0.5pmols of RNaseP probe. RT-qPCR was performed in Quantstudio 7 Flex real-time PCR machine (Thermofisher) in 96-well plates using the following program: 50^o^ 30 mins (RT), 95^o^ for 3 mins followed by 45 cycles of 95^o^ for 10 secs and 56^o^ for 30 secs. HEX and FAM signals were acquired in the 56^o^ step. After completion, the data were analyzed by setting the thresholds to 0.1 for Sgene and 0.2 for RNaseP amplification curves, respectively. Any sample with an amplification curve for the Sgene that crossed the threshold was called “Detected”. If the Sgene did not amplify and the RNaseP amplified with a Ct value of < 32, the sample was called “Not detected”. If the Sgene did not amplify and the RNaseP also failed to amplify or if the RNaseP Ct value was >32, the sample was called “Invalid”. Protocols to prevent contamination were followed throughout the workflow. Positive and negative controls were included in each run. The analytical sensitivity of the assay is 480 copies/ml of SARS-CoV-2 (95% detection frequency). The assay amplifies only the Sgene of SARS-CoV-2. The specificity of amplification was determined by spiking other respiratory viruses into saliva and by sequencing specimens with positive results. The positivity rate of SARS-CoV-2 amongst the patients in the orthodontic clinic was compared to the rate of positivity of the Chicago and Cook County databases on a daily basis.

### Positivity rate of SARS-CoV-2 infection of Chicago and Cook County, Illinois

The positivity rates of COVID-19 in Chicago and Cook County, Illinois, were reported on the webpage of Chicago: COVID-19 dashboard https://www.chicago.gov/city/en/sites/covid-19/home/covid-dashboard.html and Cook County COVID-19 Surveillance Data webpage https://ccdphcd.shinyapps.io/covid19/. The data were updated daily, and the positivity rate of SARS-CoV-2 at the orthodontic clinic, the University of Illinois Chicago was calculated from the percentage of the number of positive cases from the total number of the tests on a specific day.

### Statistical analysis

The positivity rates were reported as the percentage of the positive cases out of the total tested cases. To investigate positivity rate association among Cook County, Chicago and UIC, based on the sample activity from June, 14 to October, 29, 2021, a time-series graph and their respective cross-correlation coefficients were calculated using the software IBM Corp. Released 2021. IBM SPSS Statistics for Windows, Version 28.0. Armonk, NY: IBM Corp.

## Results

### Demographic profiles were similar between UIC and the local community

**Fig 1:**
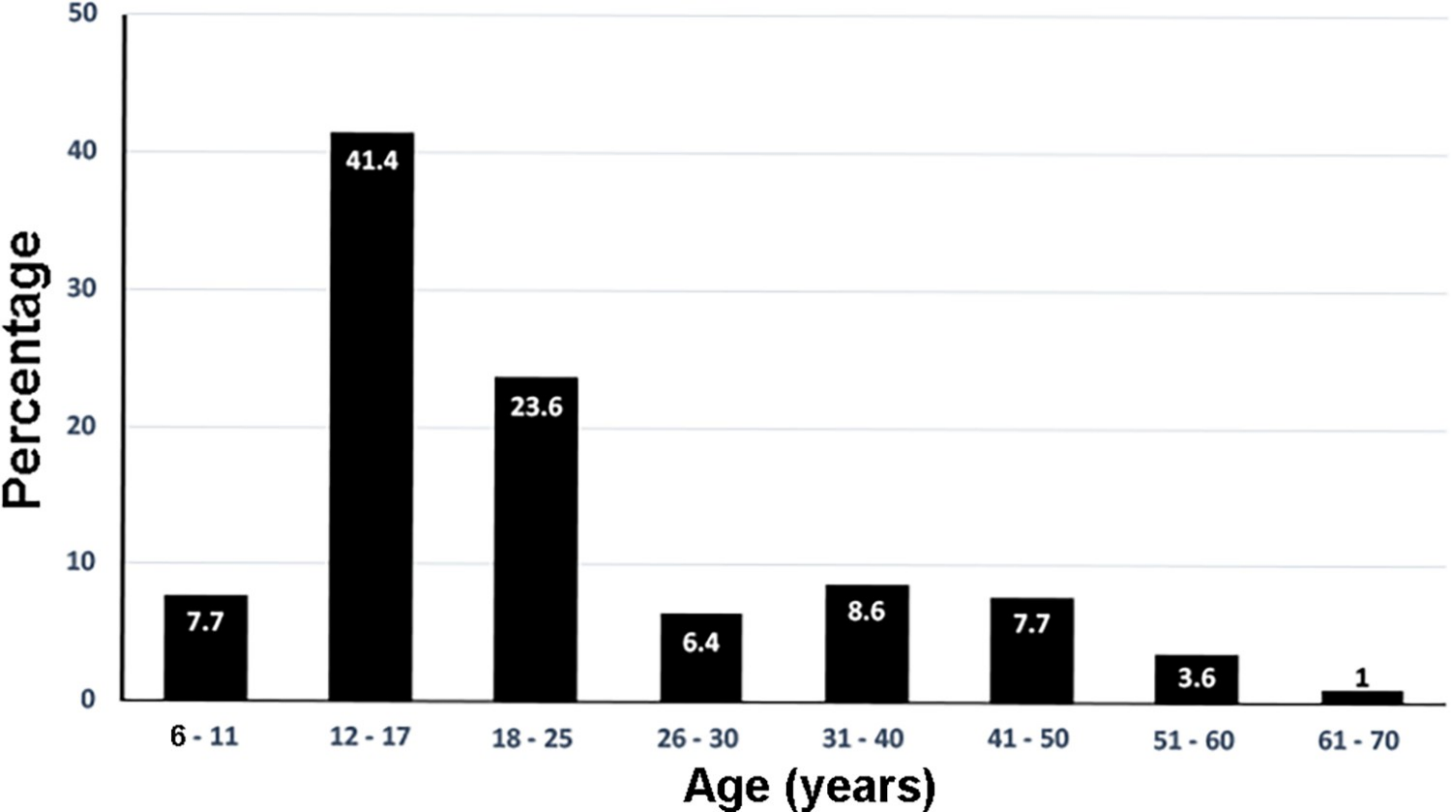
Distribution of ages of participating participants. The distribution of ages of participants is shown in Fig 1 as 65% of the participants were aged from 12–25 years old.

**Fig 2:**
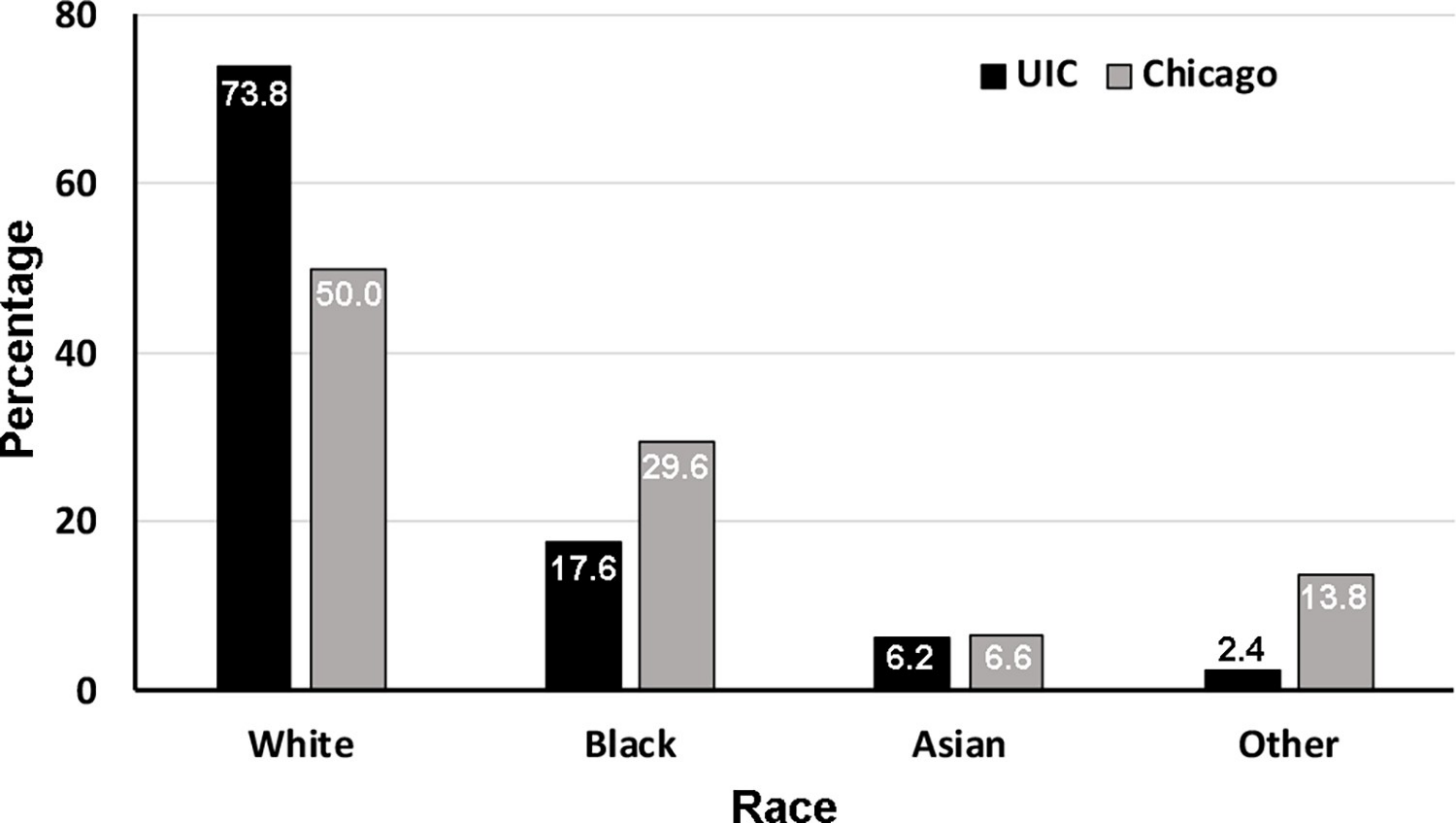
Comparison of the population by race between the patients at UIC orthodontic clinic and City of Chicago. The similar distribution of races between the orthodontic patients at UIC and the population of the City of Chicago was shown. The majority of the patients were white. Black bars: UIC; Gray bars: City of Chicago.

**Fig 3:**
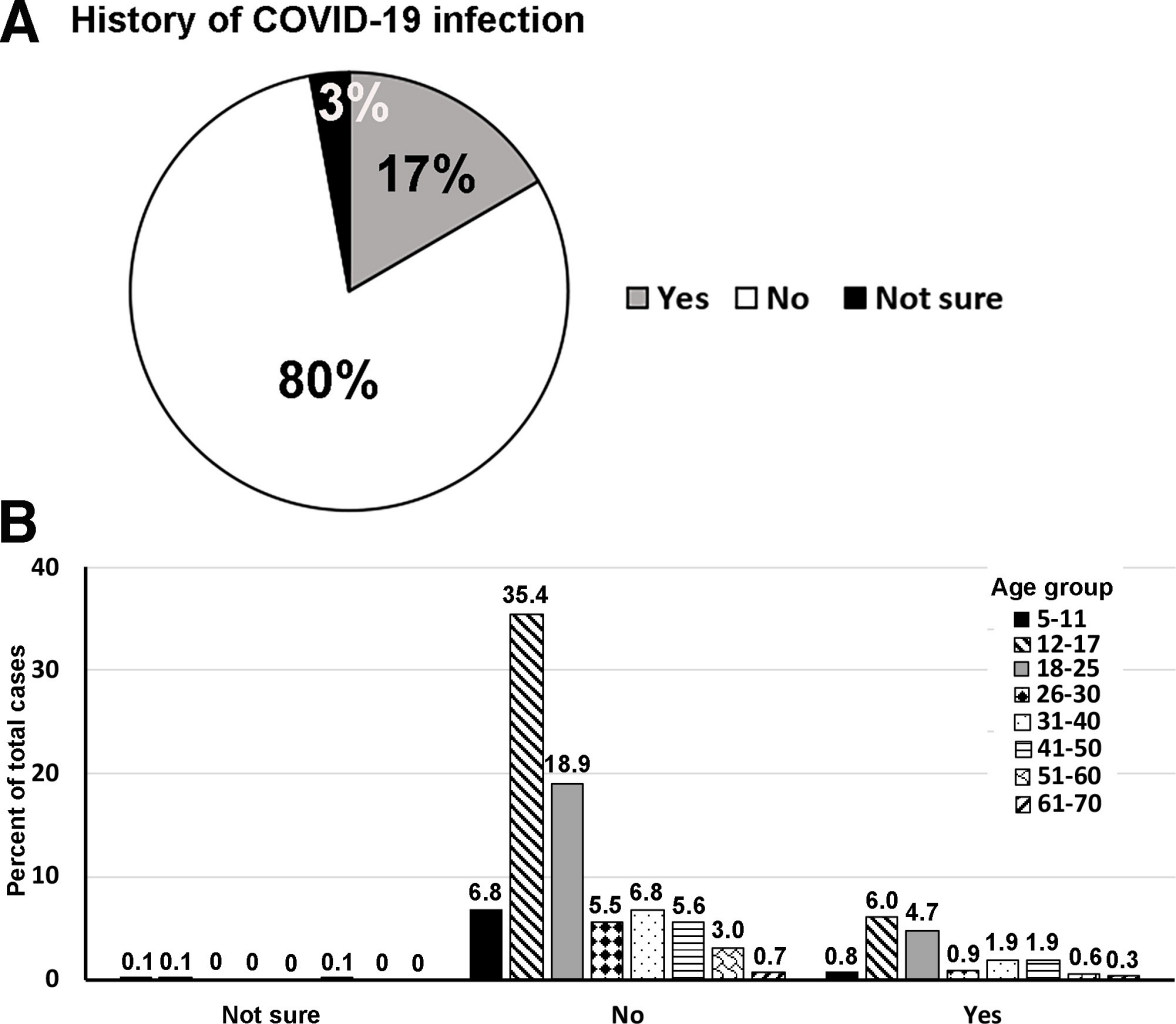
**Distribution of participants by a history of COVID-19 infection.** Most participants did not have COVID-19 infection, and only 17% of total participants had COVID-19 infection (A). The history of COVID-19 infection by age group was shown, and the group of 12–17 years old had the most number of participants with a history of COVID-19 infection (B).

**Fig 4:**
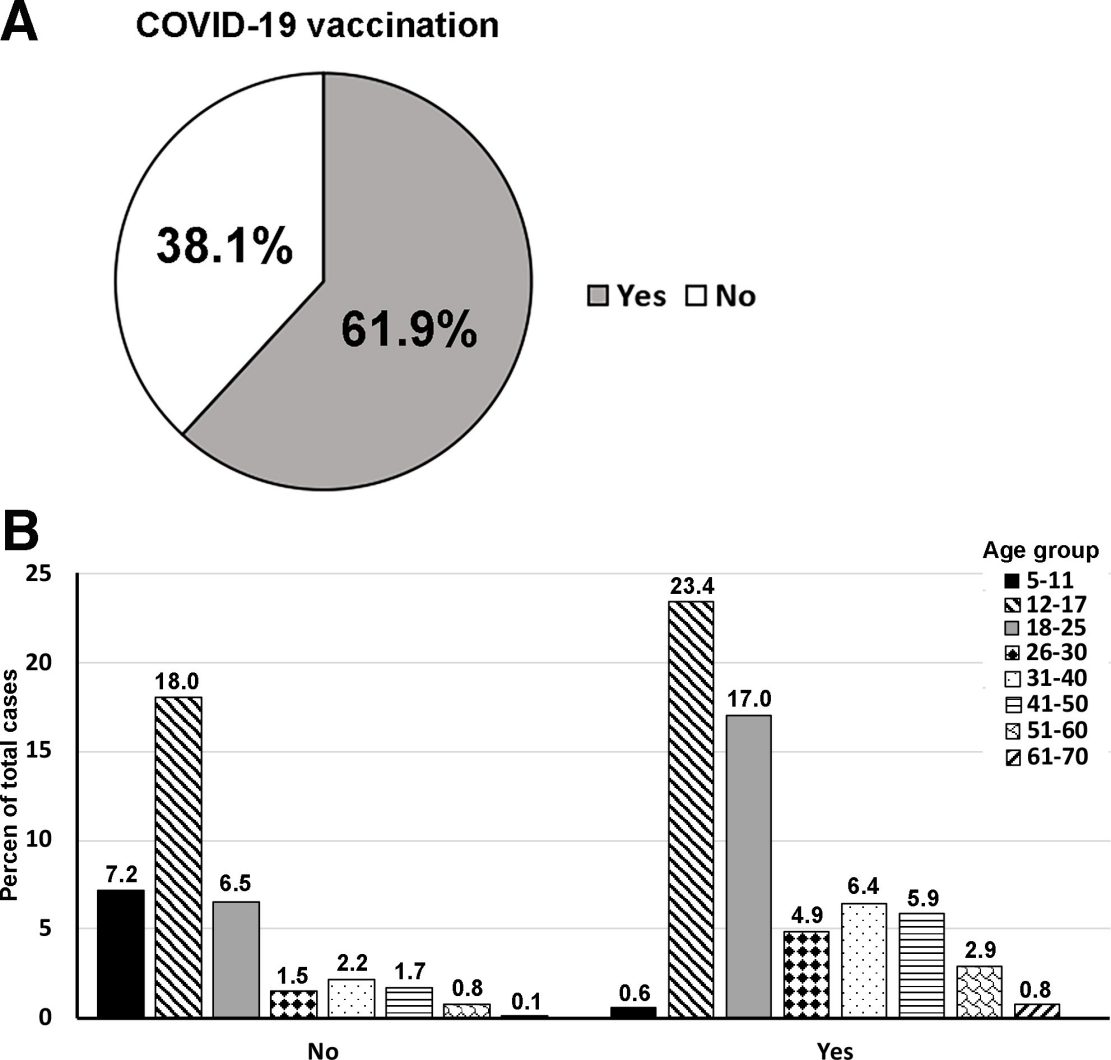
Distribution of participants by COVID-19 vaccination status. Approximately 62% of participants received full doses of COVID-19 vaccination (A). The vaccination status by age groups showed the highest number of the vaccinated group was aged 12–17 years old (B).

One thousand four hundred and thirty-seven orthodontic patients ranged from 6–70 years old, with 41.8% males and 58.2% females (Table 1) participating in the study from June 16 to October 31, 2021. The distribution of ages of participants is shown in Fig 1, as 65% of the participants were aged from 12–25 years old. The comparison of the population by race between the patient at the UIC orthodontic clinic and Chicago is shown in Fig 2. None of the participants showed any symptoms related to COVID-19 and had no history of being in contact with COVID-19 patients. The participants and guardians completed the questionnaires related to the history of COVID-19 infection and vaccination. Among all participants, 17% reported a history of COVID-19 infection (Fig 3A), and about 10.7% out of 17% were aged between 12–25 years old (Fig 3B). Among all participants, 61.9% received complete doses of COVID-19 vaccination (Fig 4A), and approximately 40% were age group between 12–25 years old (Fig 4B). Among all participants, SARS-CoV-2 was detected in nine participants (4 females and 5 males, aged 10–44 years old). All of the positive participants were asymptomatic, and two of the participants had a history of COVID-19 infection. Three positive participants had received complete COVID-19 vaccination (Table 2).

**Table 1:** Distribution of sex among the participating participants.

		Frequency	Percent
**Sex**	**Male**	601	41.8
	**Female**	836	58.2
	**Total**	1437	100

**Table 2:** Details of the SAR-CoV-2 positive participants.

Participant	Sex	Age	Race	History of infection	COVID-19 Vaccination
1	F	11	white	no	no
2	M	10	black	no	no
3	F	31	white	no	no
4	M	26	white	yes	no
5	M	10	white	no	no
6	M	13	white	yes	yes
7	M	44	white	no	yes
8	F	17	black	no	no
9	M	17	black	no	yes

### There was no correlation between the positivity rates of COVID-19 infection at the orthodontic clinic and the ones of the local community

**Fig 5:**
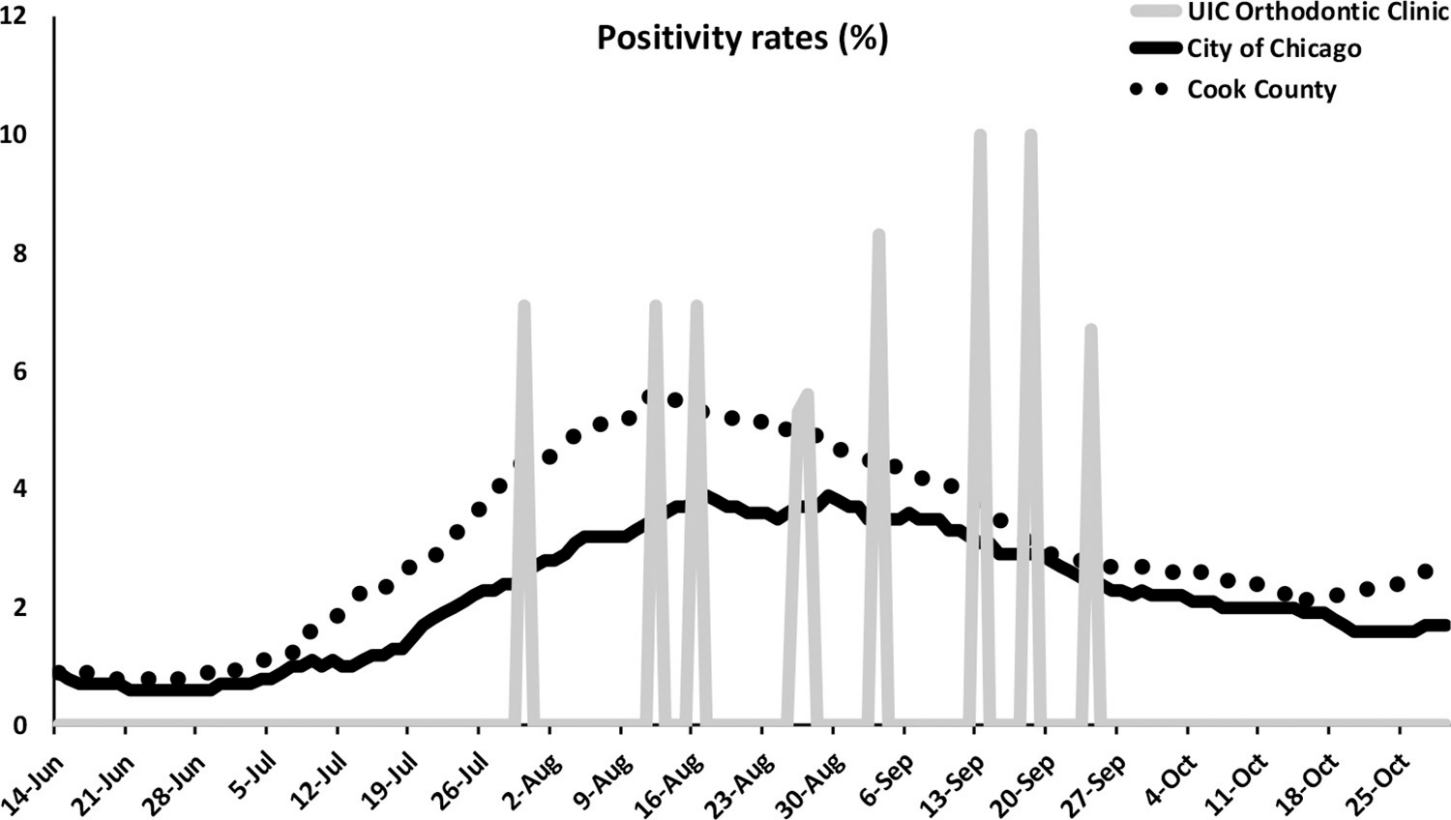
Correlations among the SARS-CoV-2 positivity rates of the orthodontic clinic, University of Illinois Chicago, Chicago and Cook County, Illinois. There was no correlation between the positivity of SARS-CoV-2 infection at the orthodontic clinic and the city of Chicago and Cook County, IL. There was a high correlation between the positivity rates of the City of Chicago and Cook County. The lag-zero correlation was 0.943, and the direction of the correlations was positive.

The average positivity rate of SARS-CoV-2 infection during the study was 0.626%. There was no correlation between the positivity of COVID-19 infection at the orthodontic clinic and the one of Chicago and Cook County, IL. The r^2^ indicates approximately 89% of the positivity rate variability in Cook County and Chicago. The cross-correlation between the positivity rates of Cook County and Chicago was statistically significant. The lag-zero correlation was 0.943, and the direction of the correlations was positive. However, the lag-zero cross-correlations positivity rates of Cook County or City of Chicago and the orthodontic clinic at UIC are very low as 0.212 and 0.240, respectively. Interestingly, the frequency of COVID-19 detection at the orthodontic clinic increased during the high positivity rate in Chicago and Cook County, IL (Fig 5). Note that Chicago posted an indoor mask mandate on August 26, 2021.

## Discussion

This study is the first to report the positivity rate of SARS-CoV-2 infection in the orthodontic patient population. Positivity rate is a better indicator of the spread of the disease than the number of confirmed cases because it is calculated from the total numbers of the employed test [32]. High positivity rate could be contributed by the high number of positive tests or the low number of total tests. The age distribution of the participants in this study were similar to the orthodontic patients’ age distribution in the United States from 1996 to 2016 [33], implicating increased numbers of the adult population. The distribution of races of the participants were similar to the distribution of races of the population in the city of Chicago as well [34]. Few reports showed that the positivity rates of SARS-CoV-2 infection in asymptomatic dental patients in Glasgow and Israel were 0.5% [35] and 0.027% [36], respectively. The positivity rate of SARS-CoV-2 infection in asymptomatic pediatric dental patients was reported as 2.3% [37] and the emergency dental department in Chicago was 6.7% [38]. However, all previously reported positivity rates were reported before the emergency usage authorization of COVID-19 vaccines [39]. Our study reported the positivity rate of SARS-CoV-2 infection in the orthodontic patient population after the COVID-19 vaccine distribution in the United States. This report represented the population with a contemporary background of COVID-19 vaccination and similar age distribution of orthodontic patients in the United States. The study utilized a highly sensitive and specific RT-qPCR test developed by the clinical diagnostic laboratory at the UIC Department of Pathology to detect SARS-CoV-2. The clinical diagnostic laboratory at the UIC Department of pathology is the Clinical Laboratory Improvement Amendments of 1988 (CLIA) and the College of American Pathologists (CAP)-accredited laboratory. During the study, the city of Chicago administered COVID-19 vaccination an average of 5,200 cases a day [40]. We collected the history of COVID-19 infection and COVID-19 vaccination status to investigate the possibility of reinfection and breakthrough infection. In this study, most participants who received complete vaccination aged between 12–25 years old. No COVID-19 vaccine was authorized to be administered to children below 11 years old during the study period. In this study, two positive participants reported a history of COVID-19 infection, indicating their reinfection status without any symptoms. There were several case reports of COVID-19 reinfection in the literature [41–43]. Three positive participants reported a history of complete COVID-19 vaccination, indicating breakthrough infection. Literature reported COVID-19 breakthrough infection in many countries, including the United States [44–46]. In this study, we did not find a statistical correlation between the daily positivity rates of SARS-CoV-2 infection at the orthodontic clinic at the University of Illinois Chicago and the city of Chicago or Cook County, IL. It is possible that the low numbers of positive cases were detected in the orthodontic patient population due to the prescreening process before the appointments. This prescreening process was implemented at the orthodontic clinic following the guidelines issued by the US CDC to implement COVID-19 mitigation for dental procedures to prevent in-office transmission [47]. Another speculation is that only asymptomatic patients sought orthodontic treatment, while the symptomatic patients would isolate themselves at home until they recovered and came back for their orthodontic appointments according to the guidelines of the US CDC. More than half of the participants had received the COVID-19 vaccines, which would affect the positivity rate of the orthodontic patients as well. The high positivity rate of Chicago and Cook County, IL were most likely obtained from the individuals who were tested when they likely had symptoms related to COVID-19.

We chose saliva as the specimen type for detecting SARS-CoV-2 in this study. The analytical sensitivity and specificity of viral detection in saliva has been reported to be equivalent or even better than NP swabs in many studies [18,48–51]. Saliva is very advantageous over NP swabs because it can be self-collected in a non-invasive manner and transported to the lab without any collection medium. Unlike NP swabs, there is no need for trained personnel to collect saliva, less painful and more subject-friendly. It is challenging to compare results from saliva and NP swabs directly because most RT-PCR tests that are performed on NP swabs are optimized to this specimen type only. Using the same test conditions for saliva may not be appropriate because unlike NP swabs, RNA extracts from saliva contain significant amounts of host cellular DNA, RNA and bacterial nucleic acids. Differences in the composition of the extracted nucleic acid may translate to differences in test performance if the test is not optimized and validated properly to detect SARS-CoV-2 in saliva. The test that we used in this study was appropriately validated in the clinical molecular pathology lab to detect SARS-CoV-2 in saliva. Saliva has also been reported to be positive for longer periods of time with higher viral loads when compared to NP swabs [52]. Overall current evidence suggests that after appropriate validation, saliva is an excellent specimen type to detect SARS-CoV2 with high sensitivity and specificity by RT-PCR tests.

## Conclusion

The positivity rate of SARS-CoV-2 infection in patients who visited the orthodontic clinic at UIC was low compared to the positivity rate in the City of Chicago and Cook county. However, our study shows that the risk of transmission to providers exists even when the positivity rate is low from individuals who are asymptomatic and vaccinated yet infected. Testing for SARS-CoV-2 on the day of the appointment may help to reduce that risk. The absence of symptoms or illness cannot be relied upon to assess COVID-19 infection status. As such, universal precautions have to be followed to mitigate the risk of transmission to providers.

## Supporting information

S1 Table Data set of PCR result from individual subject with demographic data and history of SARS-CoV-2 infection and vaccination. (XLSX)

S2 Table Data set of positivity rate of the department of Orthodontics at UIC and the rates of Chicago and Cook county. (XLSX)
